# Caudal Anesthesia for Pediatric Subumbilical Surgery, Less Load on the Postoperative Recovery Unit

**DOI:** 10.7759/cureus.4348

**Published:** 2019-03-30

**Authors:** Ruslan Abdullayev, Ulku Sabuncu, Öznur Uludağ, Hatice Selcuk Kusderci, Mesut Oterkus, Aysel Buyrukcan, Mehmet Duran, Mehmet Bulbul, Hasan Ogunc Apaydin, Nail Aksoy, Musa Abes

**Affiliations:** 1 Anesthesiology and Reanimation, Marmara University School of Medicine, İstanbul, TUR; 2 Anesthesiology and Reanimation, Tepecik Research and Educational Hospital, Izmir, TUR; 3 Anesthesiology and Reanimation, Adiyaman University Educational and Research Hospital, Adıyaman, TUR; 4 Anesthesiology and Reanimation, Bandirma State Hospital, Balikesir, TUR; 5 Anesthesiology and Reanimation, Kafkas University Medical Faculty Hospital, Kars, TUR; 6 Anesthesiology and Reanimation, Kusadasi State Hospital, Izmir, TUR; 7 Anesthesiology and Reanimation, Adiyaman University Educational and Research Hospital, Adiyaman, TUR; 8 Obstetrics and Gynecology, Adiyaman University Educational and Research Hospital, Adiyaman, TUR; 9 Pediatric Surgery, Adiyaman University Educational and Research Hospital, Adiyaman, TUR; 10 Pediatric Surgery, Kafkas University Medical Faculty Hospital, Kars, TUR

**Keywords:** caudal anesthesia, caudal block, pediatric anesthesia, general anesthesia, local anesthesia, recovery unit

## Abstract

Introduction

Caudal epidural anesthesia, when used as a sole method for surgical anesthesia, has favorable effects on the recovery duration and the time spent in the recovery unit. In this study we made a retrospective analysis of pediatric surgery operations under local, regional and general anesthesia. We aimed to find shorter postoperative recovery times with local and regional anesthesia.

Materials and methods

Data of the pediatric patients undergone subumbilical surgery during the two-year period in Pediatric Surgery clinic were collected. The patients’ age, sex, surgery type, anesthesia and airway control routes, as well as duration of anesthesia, operation and recovery were obtained.

Results

Data of 937 patients were analyzed, of whom 811 (86.6%) were males. Caudal anesthesia was performed in 240 patients (25.6%) and the mean age of these patients was 3.83 ± 3.00 years. The patients with caudal and local anesthesia spent significantly less time in the postoperative recovery unit, compared with general anesthesia groups (P < 0.001).

Conclusion

Caudal anesthesia as a sole method for pediatric subumbilical surgery is a relatively safe method. Patients having operation under caudal anesthesia have faster discharge times from postoperative recovery units, compared with general anesthesia. This probably reduces recovery unit expenditures.

## Introduction

Caudal epidural block is one of the most commonly applied regional anesthetic techniques in pediatric population. It is widely used either for anesthetic or analgesic purposes in lower abdominal and lower extremity surgeries [1,2]. First described in 1933, caudal block can be used as a sole method for surgical anesthesia, or in combination with general anesthesia to provide good postoperative analgesia [3-5]. Used as a sole method for surgical anesthesia, caudal block has advantages like avoiding volatile anesthetic agents and neuromuscular blockers, preserving spontaneous ventilation and providing early recovery. When used as adjunct to general anesthesia, it helps sparing volatile anesthetic agents and neuromuscular blockers, provides excellent analgesia. Adrenocorticotropic hormone (ACTH), beta-endorphin, antidiuretic hormone (ADH), cortisol, prolactin, and glucose levels are less affected during surgery under caudal anesthesia, compared with general anesthesia [6,7].

The block is generally performed in left lateral decubitus position with the upper hip flexed 90º and the lower 45º. Meticulous adherence to cleaning and disinfectioning protocols should be made because of the anatomic neighborhood of the area. Epidural puncture is achieved in the most proximal region of the sacral hiatus with the needle inclined 45-60º to the skin. After perforation of the membrane occluding the sacral hiatus, the needle should only be minimally advanced, not more than 1-3 mm, in order to avoid a bloody puncture or an intrathecal injection [8]. Different needles can be used for the procedure, like special caudal needle, intravenous cannula, “butterfly” needle; the main issue that should be kept in mind is the theoretical possibility of spreading of epidermal cells into the spinal canal [8].

Caudal anesthesia is a remarkably safe method [9], but serious complication can be encountered, like intrathecal or intravascular injection, resulting in total spinal block and systemic toxicity, respectively. Other complications include block failure, subcutaneous infiltration, intraosseous injection, rectal puncture, and myonecrosis [10].

When caudal anesthesia is used as a sole method to provide surgical anesthesia, it helps sparing general anesthesia and prevents side effects of drugs used, including prolonged recovery. Time required for the patient’s recovery and the postanesthesia recovery unit expenditures are decreased. In this study we made a retrospective analysis of pediatric surgery operations made in a two-year period, in which approximately quarter of the anesthesia procedures were caudal blocks. Our clinical observation was that patients with caudal block had faster recovery and return to normal activity, despite deep sedation used, and this was the basis of our hypothesis.

## Materials and methods

A total of 1042 patients having operation in Pediatric Surgery clinic of Adiyaman University Research and Educational Hospital between January 2012 and December 2013 were screened after the Investigational Review Board approval (Adıyaman Üniversitesi Biyomedikal Araştırmalar Etik Kurulu, 18.04.2014, 57831858/46). Data consisting of the patients’ age, sex, surgery type, anesthesia and airway control routes, as well as durations of anesthesia, operation and recovery were collected from the patient anesthesia charts.

We adhere to strict protocols regarding caudal epidural block in children and infants. Patients receive 0.05 mg kg-1 iv midazolam for sedation in the preoperative period if has intravenous line, or 0.1 mg kg-1 im midazolam if the line is not present. Caudal block is performed under deep sedation with ketamine 1 mg kg-1 iv and propofol 1 mg kg-1 iv. In rare cases inhalation anesthesia induction is done when problems with intravenous line are encountered, which is performed via 8% sevoflurane in 1:1 mixture of oxygen and nitrous oxide; and after the intravenous line is established caudal block is performed. It is avoided in patients with low body weight (<2 kg) and over 25 kg, bleeding-clotting disorders, abnormal vertebral anatomy, local infection, and fever. Standard monitoring consists of electrocardiogram, peripheral oxygen saturation, and non-invasive blood pressure. Caudal block is performed in left lateral decubitis position after appropriate disinfectioning procedures. A 24 Gauge intravenous catheter is used for puncture. Local anesthetics used for surgical anesthesia are lidocaine, prilocaine or bupivacaine diluted as 1:1 with physiologic saline, and the dose is 1 mL kg-1 body weight. This dose is our standard protocol used for subumbilical surgery.

Vast majority of the patients undergoing circumcision had local anesthesia performed by the surgeon, again under deep sedation. Either propofol 1 mg kg-1 iv or ketamine 1 mg kg-1 iv was used for that purpose. Local anesthetic agents used were lidocaine 1% or prilocaine 1% in doses 0.25 mL kg-1 body weight. Again, patients with difficult intravenous lines were subjected to the above-mentioned protocol.

If general anesthesia was to be applied either propofol 2.5 mg kg-1 iv or thiopental sodium 6 mg kg-1 iv was used for induction, followed by vecuronium 0.1 mg kg-1 iv or rocuronium 0.8 mg kg-1 iv. Airway control was provided by classical laryngeal mask airway (LMA), or endotracheal tube when neuromuscular blockers were used.

Statistical analysis was made by Statistical Package for the Social Sciences software version 13.0 (SPSS Inc., Chicago, IL, USA). Continuous variables are given as mean ± standard deviation. In comparisons for more than two groups, One-Way ANOVA test was used for data with normal distribution, and Kruskal-Wallis test was used for data without normal distribution. Groups comprising categorical variables were compared with Pearson Chi-Square test. The level of statistical significance was defined as P < 0.05.

## Results

Out of 1042 patients, data of 937 with subumblical surgeries were selected for further analysis. Patients undergoing multiple operations and children with unsuccessful caudal blocks were excluded. The patients’ operations are given in Table 1. The number of male patients was 811 (86.6%), while the rest were female.

**Table 1:** Operations performed on patients. *Herniorrhaphy was performed on patients with inguinal and umbilical hernia, and both operations were grouped together to facilitate data interpretation.

Operation	Number of operations (n = 937)	Percent
Circumcision	336	35.9
Herniorrhaphy*	270	28.8
Appendectomy	138	14.7
Hypospadias repair	62	6.6
Hydrocelectomy	60	6.4
Orchiopexy	50	5.3
Manual reduction of invagination	11	1.2
Cordon cyst excision	10	1.1

Distribution of the patients according to the operative anesthesia applied is demonstrated in Table 2. As seen in the table, caudal anesthesia was applied to 240 patients and constituted a quarter of all the operations. Difference between ages was statistically significant, and the patients having caudal block formed the youngest group with mean age of 3.83 years.

**Table 2:** Distribution of the patients regarding anesthesia application. Note: Numbers in the parentheses represent percent values of total. Age values are given as mean ± standard deviation (SD). *P < 0.001 LMA: Laryngeal mask airway.

	Number of patients (n = 937)	Age (years)	
Endotracheal intubation	272 (29.0)	8.31 ± 4.61	P < 0.001*
LMA	343 (36.6)	5.43 ± 3.89	
Caudal block	240 (25.6)	3.83 ± 3.00	
Local anesthesia	82 (8.8)	5.76 ± 4.43	

Durations of anesthesia and surgery of the patients are represented in Table 3. Anesthesia duration indicates the time from the start of anesthesia induction until the time the patient is to be transferred to the postoperative recovery unit, whereas surgery duration is the time from the start of disinfectioning until the surgical wound dressing. Difference between these times, which indicated time spent for anesthesia induction and recovery on the operating room table, was lowest in the local anesthesia group (P = 0.015), whereas the results were comparable in the other groups.

**Table 3:** Anesthesia and surgery durations of the patients. Note: Values are expressed as mean ± SD. Difference shows time difference between anesthesia and surgery durations, and indicates time spent for anesthesia induction and recovery. *P < 0.05 LMA: Laryngeal mask airway.

	Anesthesia duration (min)	Surgery duration (min)	Difference (min)	
Endotracheal intubation	63.25 ± 25.68	53.10 ± 25.46	10.16 ± 6.51	P = 0.015*
LMA	46.74 ± 14.28	37.71 ± 13.91	9.02 ± 4.33	
Caudal block	56.80 ± 24.17	47.15 ± 23.73	9.65 ± 6.06	
Local anesthesia	37.99 ± 12.47	29.63 ± 11.05	8.35 ± 2.73	

Postoperative recovery unit length of stay of the patients is represented in Table 4. The patients with caudal and local anesthesia spent significantly less time in the postoperative recovery unit (P < 0.001).

**Table 4:** Postoperative recovery unit length of stay of the patients. Note: Values are given in mean ± SD. *P < 0.001 for all intergroup comparisons, but for caudal versus local anesthesia groups, where P = 0.989. LMA: Laryngeal mask airway.

	Recovery unit time (min)	P
Endotracheal intubation	15.17 ± 3.92	< 0.001*
LMA	13.03 ± 3.22	
Caudal block	10.56 ± 2.28	
Local anesthesia	10.06 ± 1.84	

No serious adverse effects were observed in the patients, including complications of caudal anesthesia, like total spinal block or systemic toxicity. Of 246 caudal blocks performed, only six were insufficient for surgery as a sole method, and were supported with general anesthesia, i.e., unsuccessful block rate was 2.44%. As mentioned above, unsuccessful blocks were excluded from the analysis, because general anesthesia was provided for them.

## Discussion

The main finding of the study was that patients with caudal and local anesthesia had significantly shorter lengths of stay in the postanesthesia care unit. The statistically significant difference may not be so important clinically, as we deal with few extra minutes spent in recovery unit, but taking into consideration that pediatric population is special, and an extra nurse is assigned for each patient, the difference gains more importance. The mean age of the patients was least in the group with caudal blocks, and this reflects the preference of anesthesiologists because of anatomical considerations of this age. Time spent for anesthesia application was comparable between the groups, probably indicating more preoperative time for caudal and more postoperative time for general anesthesia in operating room.

Caudal block is known for more than 80 years, and complications are also well recognized, including dural puncture, intravascular injection, rectal penetration, drug overdose, morphine apnea. Several techniques have been considered in detecting proper placement of caudal needle in the epidural space, including nerve stimulation, ultrasound imaging, whoosh test, and modified swoosh test [11-13]. Still some new application techniques are evolving to minimize complication rates [14,15]. Although aspiration and return of blood or liquor is definite evidence for intravascular or intrathecal needle misplacement, a negative aspiration lacks sensitivity in excluding these complications. The most important method of identifying accidental systemic injection is the “test dose” with epinephrine, although it lacks sensitivity for intrathecal misplacement [16,17].

Many adjuvant drugs have been used along with local anesthetics in caudal block to prolong postoperative analgesia. They include clonidine, fentanyl morphine, neostigmine, ketamine, midazolam, and tramadol [18-20]. Caudal block decreases postoperative systemic opioid need, but if opioids are used as adjuncts to local anesthetics side-effects should be kept in mind, especially late respiratory depression with morphine. The use of opioids was superseded by non-opioids for these reasons, but they also have some side effects. Neostigmine is associated with high incidence of nausea and vomiting. Some debate exists about possible neurotoxic effects of ketamine. Effects of clonidine and midazolam remain controversial. Cognizance should be taken of the fact that these drugs are not licensed for use in the epidural space [17]. Epidural catheters are not recommended because of anatomical neighborhoods and high risk of infection.

Postoperative recovery time indicates the length of time elapsed from the end of surgery until a patient is deemed ready for discharge after surgery. This time varies among the patients and is influenced by anesthetic technique, and often used as a measure of cost-effectiveness for anesthetics [21]. Many of the features of regional anesthetic techniques make it the ideal, cost-effective outpatient anesthetic [22]. The use of regional anesthesia for surgery provides anesthetists the ability to reduce, and even eliminate, the need for parenteral opioids along with opioid-induced side effects like nausea, vomiting, respiratory depression, common causes of delayed discharge from postanesthesia recovery units [23]. The improved patient outcomes experienced in the recovery room lend themselves to increased nursing efficiency, as fewer interventions are necessary, thereby reducing system factor delays and freeing up postanesthesia care unit resources [18]. Aldrete recovery score is used to evaluate suitability of the patients to be discharged. It is based on five criteria: activity, respiration, circulation, consciousness and color; and scores of nine or ten (the maximum) are eligible with safe discharge [24]. The approach in our institution is to assign a separate nurse for each pediatric patient in postanesthesia care unit, to increase the quality of follow-up and prevent complications like falling from the bed. As this complication is generally not a concern for adult patients, each freed nurse can deal with the follow-up of at least three adult patients.

Some special issues must be taken into account when applying caudal blocks. Micturition impairment and urinary retention are of quite importance, and the incidence is estimated to be 0.7-5.3% [25,26]. Metzelder et al. have recommended penile block instead of caudal for hypospadias repairs, where spontaneous postoperative micturition must be guaranteed [27]. Caudal block can result in intraoperative penile engorgement, because of vasodilatation and pooling of blood in the venous sinuses of the penis. This can result in oozing in surgical site together with placing surgical sutures under tension and improper healing. Penile block is again superior in these instances [28]. Motor block and delayed walking up to six hours can be encountered, and this can prolong hospital stay despite short postanesthesia recovery unit stays [29]. And finally, perhaps the most important, the doses of local anesthetic agents used are very close to maximum doses allowed, so meticulous calculations must be made during drug preparations, especially when the block is going to be used as a sole method for surgical anesthesia [30].

There have been some limitations of the study, the main of which is the retrospective nature. Data about minor complications like subcutaneous infiltration were absent, as were data about postoperative complications like nausea, vomiting, delayed walking, and micturition delay. It was not possible to obtain from the charts of Aldrete recovery scores of all the patients; this would have given more objective data regarding patient discharge times.

## Conclusions

In conclusion, caudal anesthesia as a sole method for pediatric subumbilical surgery is a relatively safe method. Patients with caudal blocks have faster discharge times from postoperative recovery units, compared with general anesthesia.
